# Supplementary material for: Characterization of Immunoactive and Immunotolerant CD4+ T Cells in Breast Cancer by Measuring Activity of Signaling Pathways That Determine Immune Cell Function
Source: Cancers (Basel). 2022 Jan 19;14(3):490. doi: 10.3390/cancers14030490 (PMC8833374; doi:10.3390/cancers14030490)
Supplement: Supplementary file 1 [file cancers-14-00490-s001.zip › cancers-1536400-supplementary.pdf]

SUPPLEMENTARY INFORMATION

sample category	annotation per sample	FOXO	NFκB	JAK-STAT1/2	JAK-STAT3	TGFβ	Notch	ER	WNT
CD4+ T cells from peripheral blood	donor 1	4.5	4.5	-8.3	-5.7	-13.9	-8.9	-18.7	-19.1
	donor 2	1.3	1.4	-7.6	-10.2	-16.8	-9.6	-19.1	-21.0
	donor 3	-3.2	-7.1	-7.6	-8.4	-20.3	-8.9	-18.9	-24.5
	donor 4	-2.1	-5.7	-6.1	-9.8	-19.0	-9.3	-18.9	-21.4
	patient 1	8.5	17.4	-7.4	-2.4	-5.0	1.6	-16.0	-20.0
	patient 2	-1.6	-1.5	-7.7	-7.3	-14.5	-5.7	-18.6	-16.7
	patient 3	5.9	6.3	-8.7	-5.5	-14.7	-8.7	-18.8	-22.8
	patient 4	-3.8	-5.1	-5.6	-6.2	-19.6	-6.1	-18.6	-21.1
	patient 5	2.4	8.4	-8.7	-4.3	-11.7	-4.2	-18.2	-15.9
	patient 6	1.7	5.9	-7.2	-6.3	-13.2	-8.4	-18.8	-21.2
	patient 7	6.9	3.2	-8.3	-5.8	-14.3	-7.3	-18.9	-19.2
	patient 8	1.3	5.2	-9.3	-6.7	-14.9	-6.8	-18.7	-17.0
	patient 9	-2.5	-1.3	-4.2	-8.1	-18.4	-8.0	-19.9	-22.2
	patient 10	-2.2	-3.6	-6.2	-7.3	-18.8	-8.4	-19.3	-21.8
CD4+ T cells from axillary lymph nodes	patient 1	-1.4	13.1	-9.0	-6.7	-12.3	-2.2	-18.8	-22.7
	patient 2	2.5	11.0	-8.3	-2.9	-11.5	0.8	-17.5	-20.8
	patient 3	5.5	17.8	-8.6	-4.5	-12.2	0.3	-18.2	-20.5
	patient 4	0.8	10.1	-8.2	-4.8	-11.7	-3.0	-18.2	-21.7
	patient 5	4.2	7.4	-6.6	-7.4	-13.2	-5.4	-18.7	-20.7
	patient 6	-3.5	-2.1	-10.5	-4.6	-15.6	-8.3	-19.9	-23.4
	patient 7	2.1	12.4	-9.0	-3.1	-10.4	-3.3	-18.2	-20.3
	patient 8	0.6	7.2	-9.4	-4.8	-14.6	-5.1	-18.7	-26.9
	patient 9	-4.0	5.6	-5.7	-5.2	-17.2	-8.6	-19.1	-25.0
	patient 10	2.3	2.9	-8.1	-3.0	-14.3	-4.0	-19.1	-21.9
CD4+ T cells from breast tumor	patient 1	5.6	21.5	-6.3	-3.9	-7.5	6.3	-18.5	-21.5
	patient 2	6.9	26.8	0.5	1.2	-6.8	2.5	-15.9	-17.5
	patient 3	10.9	30.3	-1.2	0.9	-2.4	9.3	-16.9	-15.3
	patient 4	0.9	23.5	0.9	0.3	-8.5	1.5	-15.7	-19.9
	patient 5	7.2	31.3	-4.0	5.4	-4.8	7.0	-6.7	-14.2
	patient 6	9.6	19.0	-9.8	-3.3	-10.4	4.0	-19.3	-16.6
	patient 7	5.4	22.8	-1.3	0.4	-9.0	5.6	-18.3	-13.5
	patient 8	3.2	13.6	-4.5	-3.2	-8.3	5.2	-17.5	-25.4
	patient 9	-1.9	18.1	-3.4	-4.2	-14.2	-1.3	-17.6	-25.0
	patient 10	14.5	24.8	-2.2	1.3	-7.4	9.3	-18.2	-19.1

Supplementary Figure S1. Signaling pathway activities per patient from dataset GSE36765 [19].

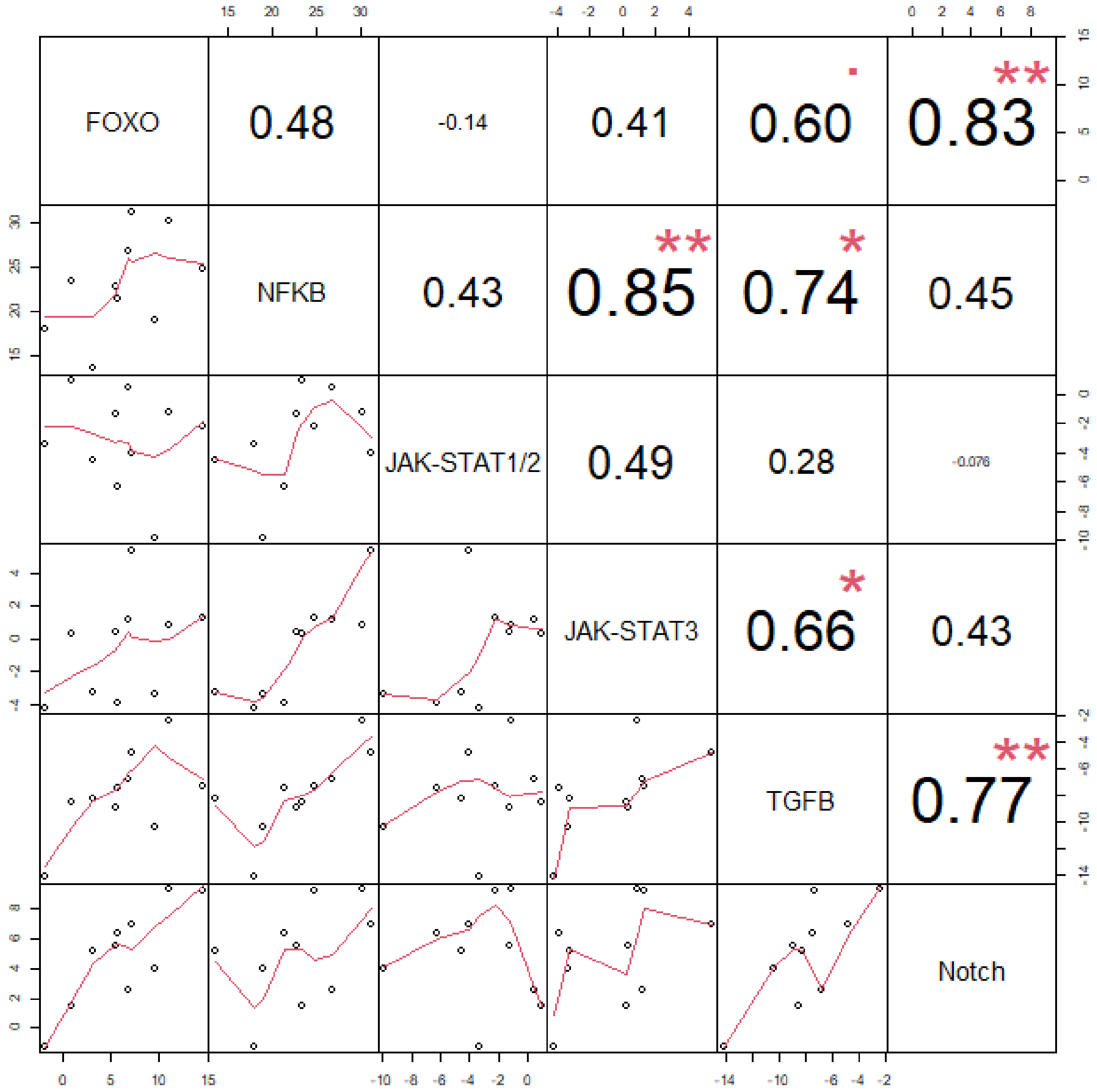

Supplementary Figure S2. NFκB pathway activity is highly correlated with JAK-STAT3 and TGFβ pathway activity. Correlation matrix between signaling pathway activities among BrCa TIL samples from dataset GSE36765 [19]. Bottom graphs show bivariate scatterplots with a fitted line and on top are the corresponding Pearson correlation coefficients, including starts for statistically significant correlations.

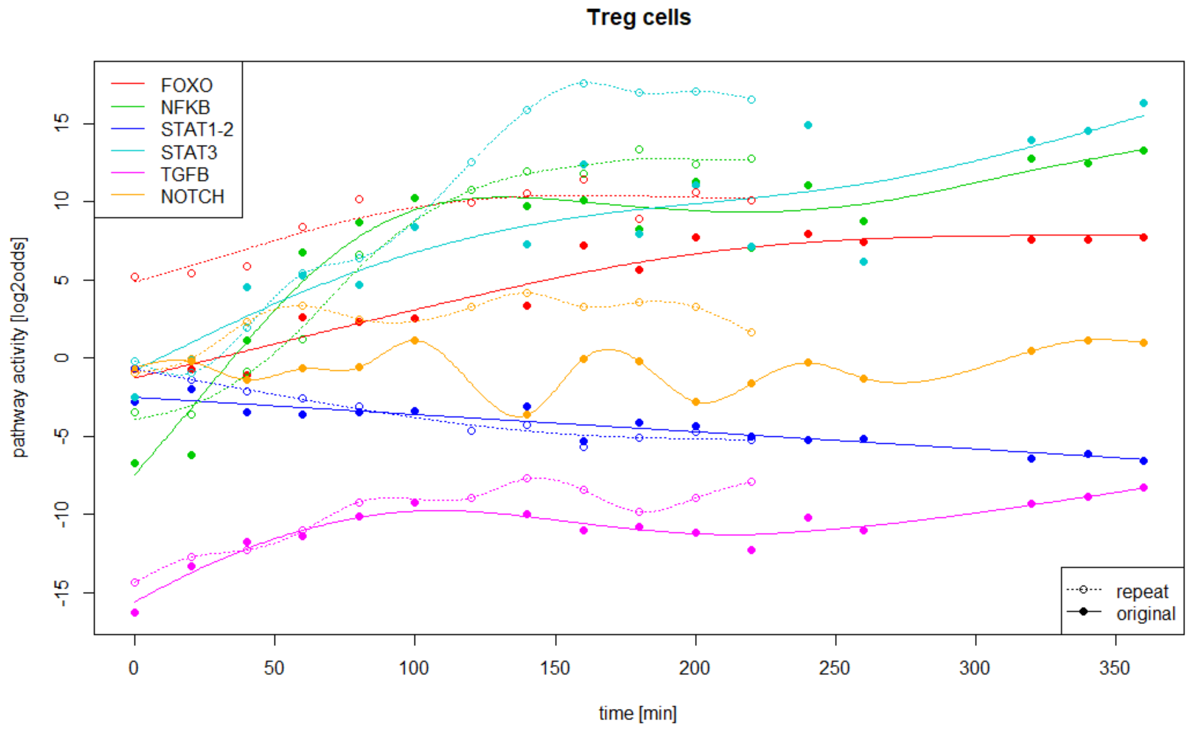
Treg cells
FOXO
NFKB
STAT1-2
STAT3
TGFB
NOTCH
repeat
original
pathway activity [log2odds]
time [min]
-15
-10
-5
0
5
10
15
0
50
100
150
200
250
300
350

A

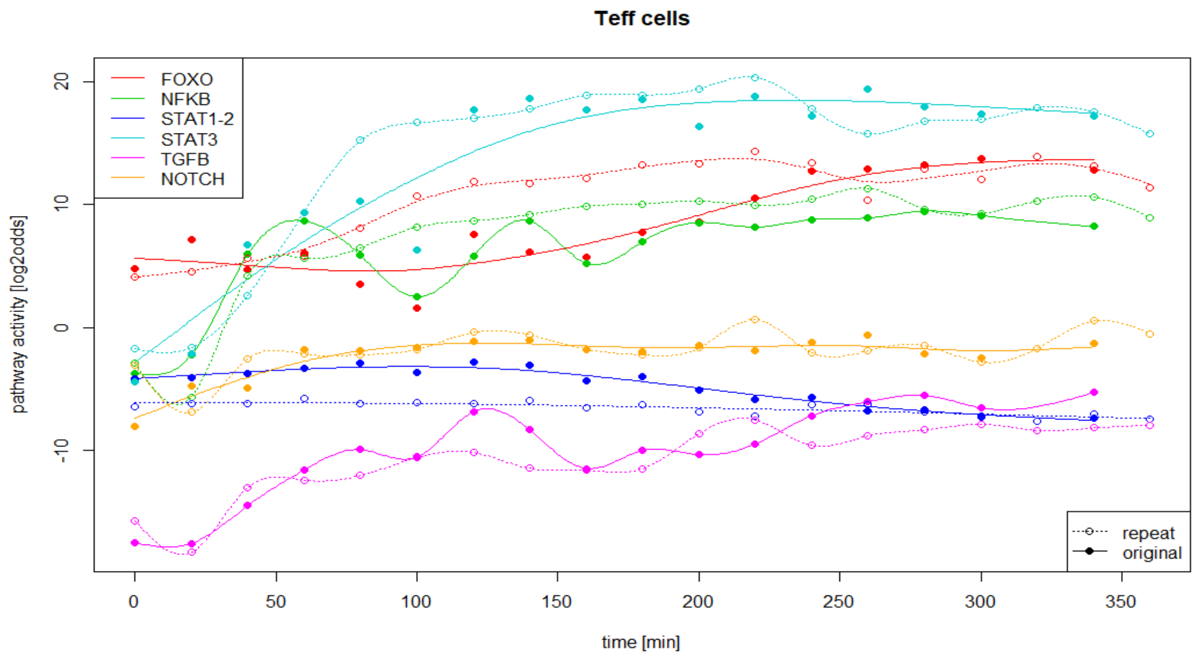
Teff cells
FOXO
NFKB
STAT1-2
STAT3
TGFB
NOTCH
repeat
original
pathway activity [log2odds]
time [min]
-10
0
10
20
0
50
100
150
200
250
300
350

B

C

array	title	QC.passed	QC.man. passed	t	log2dds NOTCH	log2odds JAK-STAT3	log2odds NFκB	log2odds JAK-STAT1/2	log2odds TGFβ		QC.man.comments
GSM285027	Human_Treg_T0min	TRUE	TRUE	0	-0.6	-2.5	-6.7	-6.3	-16.3		
GSM723815	Human_Treg_repeated_T0min	FALSE	TRUE	0	-0.9	-0.2	-3.4	-4.0	-14.3		samprep probably not performed
GSM285028	Human_Treg_T20min	FALSE	TRUE	20	-0.2	-0.1	-6.2	-5.9	-13.3		hybspike probably not performed
GSM723816	Human_Treg_repeated_T20min	TRUE	TRUE	20	0.0	-1.0	-3.6	-4.9	-12.7		
GSM285029	Human_Treg_T40min	FALSE	TRUE	40	-1.4	4.5	1.1	-6.6	-11.7		samprep and hybspike probably not performed
GSM723817	Human_Treg_repeated_T40min	TRUE	TRUE	40	2.3	1.9	-0.9	-5.4	-12.3		
GSM285030	Human_Treg_T60min	FALSE	TRUE	60	-0.7	5.3	6.8	-5.7	-11.4		hybspike probably not performed
GSM723818	Human_Treg_repeated_T60min	TRUE	TRUE	60	3.3	5.4	1.2	-5.7	-11.0		
GSM285031	Human_Treg_T80min	FALSE	TRUE	80	-0.6	4.7	8.6	-5.3	-10.1		gapdh a bit higher but .mid value OK (1.00)
GSM723819	Human_Treg_repeated_T80min	TRUE	TRUE	80	2.5	6.4	6.6	-5.7	-9.2		
GSM285032	Human_Treg_T100min	FALSE	TRUE	100	1.1	8.4	10.2	-5.6	-9.3		samprep probably not performed; hybspike OK
GSM723820	Human_Treg_repeated_T100min	FALSE	**FALSE**	100							SET TO FALSE! bactin (4.00), gapdh (2.60) and gapdh.mid (1.74) too high
GSM723821	Human_Treg_repeated_T120min	TRUE	TRUE	120	3.2	12.5	10.8	-5.4	-8.9		
GSM285033	Human_Treg_T140min	FALSE	TRUE	140	-3.6	7.3	9.7	-4.8	-10.0		gapdh a bit higher but .mid value OK (1.05)
GSM723822	Human_Treg_repeated_T140min	TRUE	TRUE	140	4.2	15.8	11.9	-6.3	-7.7		
GSM285034	Human_Treg_T160min	FALSE	TRUE	160	-0.1	12.4	10.1	-6.9	-11.0		samprep and hybspike probably not performed
GSM723823	Human_Treg_repeated_T160min	FALSE	TRUE	160	3.3	17.6	11.8	-6.8	-8.4		samprep probably not performed
GSM285035	Human_Treg_T180min	FALSE	TRUE	180	-0.2	7.9	8.2	-5.1	-10.8		hybspike probably not performed
GSM723824	Human_Treg_repeated_T180min	TRUE	TRUE	180	3.6	17.0	13.3	-6.6	-9.8		
GSM285036	Human_Treg_T200min	FALSE	TRUE	200	-2.8	11.1	11.3	-6.2	-11.1		hybspike probably not performed
GSM723825	Human_Treg_repeated_T200min	TRUE	TRUE	200	3.3	17.1	12.4	-6.1	-8.9		
GSM285037	Human_Treg_T220min	FALSE	TRUE	220	-1.6	7.1	7.1	-5.5	-12.3		samprep probably not performed
GSM723826	Human_Treg_repeated_T220min	TRUE	TRUE	220	1.6	16.6	12.7	-6.1	-7.9		
GSM285038	Human_Treg_T240min	FALSE	TRUE	240	-0.3	14.9	11.0	-7.1	-10.2		samprep probably not performed
GSM723827	Human_Treg_repeated_T240min	FALSE	**FALSE**	240							SET TO FALSE! bactin (5.18), gapdh (2.73) and gapdh.mid (1.83) too high
GSM285039	Human_Treg_T260min	FALSE	TRUE	260	-1.3	6.1	8.7	-6.2	-11.0		samprep and hybspike probably not performed
GSM723828	Human_Treg_repeated_T260min	FALSE	FALSE	260							bactin (5.46), gapdh (3.09) and gapdh.mid (2.04) too high
GSM285040	Human_Treg_T280min	FALSE	FALSE	280							bactin.mid too high (3.27)
GSM723829	Human_Treg_repeated_T280min	FALSE	FALSE	280							bactin (5.09), gapdh (2.73) and gapdh.mid (1.76) too high
GSM285041	Human_Treg_T300min	FALSE	FALSE	300							bactin (4.89) and bactin.mid (3.28) too high; failure of 6 ref genes
GSM723830	Human_Treg_repeated_T300min	FALSE	**FALSE**	300							SET TO FALSE! bactin (5.13), gapdh (2.51) and gapdh.mid (1.63) too high
GSM285042	Human_Treg_T320min	TRUE	TRUE	320	0.4	14.0	12.8	-7.2	-9.3		
GSM723831	Human_Treg_repeated_T320min	FALSE	FALSE	320							bactin (5.11), gapdh (2.96) and gapdh.mid (2.18) too high
GSM285043	Human_Treg_T340min	FALSE	TRUE	340	1.2	14.5	12.5	-7.3	-8.9		samprep and hybspike probably not performed
GSM723832	Human_Treg_repeated_T340min	FALSE	FALSE	340							bactin (4.98), gapdh (2.46) and gapdh.mid (1.54) too high
GSM285044	Human_Treg_T360min	FALSE	TRUE	360	1.0	16.3	13.2	-8.3	-8.3		samprep probably not performed
GSM723833	Human_Treg_repeated_T360min	FALSE	**FALSE**	360							SET TO FALSE! bactin (4.98), gapdh (2.49) and gapdh.mid (1.59) too high

Supplementary Figure S3. GSE11292. Pathway analysis scores for resting and activated Treg (A) and T effector (B) cells. C: QC analysis of individual sample data. Treg and T effector cells were activated in the same manner with anti-CD3/CD28 and IL2. Samples were taken every 20 minutes. N=2 time experiments (defined as "original" and "repeated") [24]. Sample data that failed our QC as described in Methods were not included in the pathway analysis. Pathway activity scores are on log2odds scale.

A

	Pearson correlation coefficients
**FOXO**	R = 0.23, p = 0.53
**NFκB**	R = 0.0094, p = 0.98
**JAK-STAT1/2**	R = 0.10, p = 0.77
**JAK-STAT3**	R = 0.10, p = 0.78
**TGFβ**	R = 0.28, p = 0.44
**Notch**	R = 0.055, p = 0.88

B

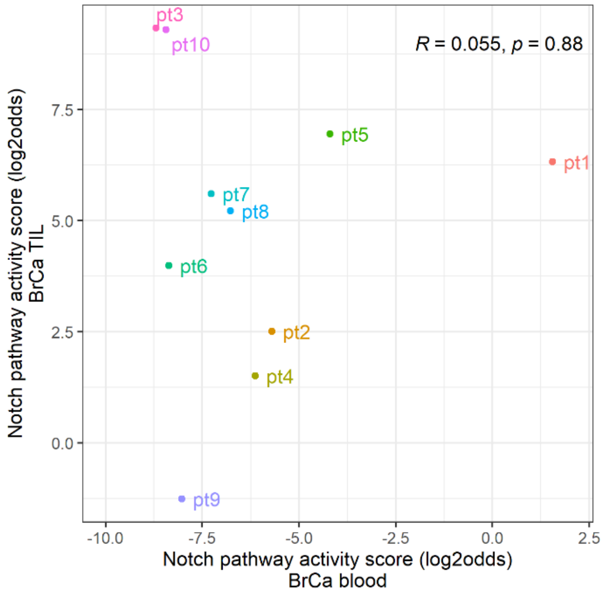

Supplementary Figure S4. Correlation between signaling pathway activities in CD4 T+ cells from blood and from TIL from BrCa patients (dataset GSE36765, [19]). A. Pearson correlation coefficients; B. Correlation plot for the Notch pathway.

Supplementary Table S1. Threshold values above which signaling pathway activity may be considered abnormally high. Thresholds are calculated based on activity in CD4+ T cells in blood from the four healthy donors in the GSE36765 dataset [19] and are defined as the mean pathway activity score ± two standard deviations.

	**lower threshold**	**upper threshold**	**# patients exceeding** the **upper threshold**
**FOXO**	-6.9	7.2	1 (pt 1)
**NFκB**	-12.8	9.4	1 (pt 1)
**JAK-STAT1/2**	-9.2	-5.5	1 (pt 9)
**JAK-STAT3**	-12.6	-4.4	2 (pt 1, 5)
**TGFβ**	-23.1	-11.9	2 (pt 1, 5)
**Notch**	-9.8	-8.5	9 (pt 1-2, 4-10)
